# Direct synthesis, characterization, *in vitro* and *in silico* studies of simple chalcones as potential antimicrobial and antileishmanial agents

**DOI:** 10.1098/rsos.240410

**Published:** 2024-06-26

**Authors:** Haroon ur Rashid, Sherwali Khan, Irum, Asad Khan, Nasir Ahmad, Tanzeel Shah, Khalid Khan

**Affiliations:** ^1^ Center for Chemical, Pharmaceutical and Food Sciences, Federal University of Pelotas, Pelotas, RS, Brazil; ^2^ Department of Chemistry, Rawalpindi Women University, 6th Road Satellite Town, Rawalpindi, Pakistan; ^3^ Department of Chemistry, Islamia College University Peshawar, Khyber Pakhtunkhwa, Pakistan; ^4^ Institute of Basic Medical Sciences, Khyber Medical University, Peshawar, Khyber Pakhtunkhwa, 25120, Pakistan

**Keywords:** chalcone, benzaldehyde, acetophenone, biomolecule, antimicrobial

## Abstract

Chalcone represents a vital biosynthetic scaffold owing to its numerous therapeutic effects. The present study was intended to synthesize 17 chalcone derivatives (**3a**–**q**) by direct coupling of substituted acetophenones and benzaldehyde. The target chalcones were characterized by spectroscopic analyses followed by their *in vitro* antimicrobial, and antileishmanial investigations with reference to standard drugs. The majority of the chalcones displayed good to excellent biological activities. Chalcone **3q** (1000 µg ml^−1^) exhibited the most potent antibacterial effect with its zone of inhibition values of 30, 33 and 34 mm versus *Staphylococcus aureus*, *Escherichia coli* and *Pseudomonas aeruginosa* respectively. The results also confirmed chalcone **3q** to be the most potent versus *Leishmania major* with the lowest IC_50_ value of 0.59 ± 0.12 µg ml^−1^. Chalcone **3i** (500 µg ml^−1^) was noticed to be the most potent antifungal agent with its zone of inhibition being 29 mm against *Candida albicans*. Computational studies of chalcones **3i** and **3q** supported the preliminary *in vivo* results. The existence of the amino moiety and bromine atom on ring-A and methoxy moieties on ring-B caused better biological effects of the chalcones. In brief, the investigations reveal that chalcones (**3i** and **3q**) can be employed as building blocks to discover novel antimicrobial agents.

## 1. Introduction

**Figure 1.**
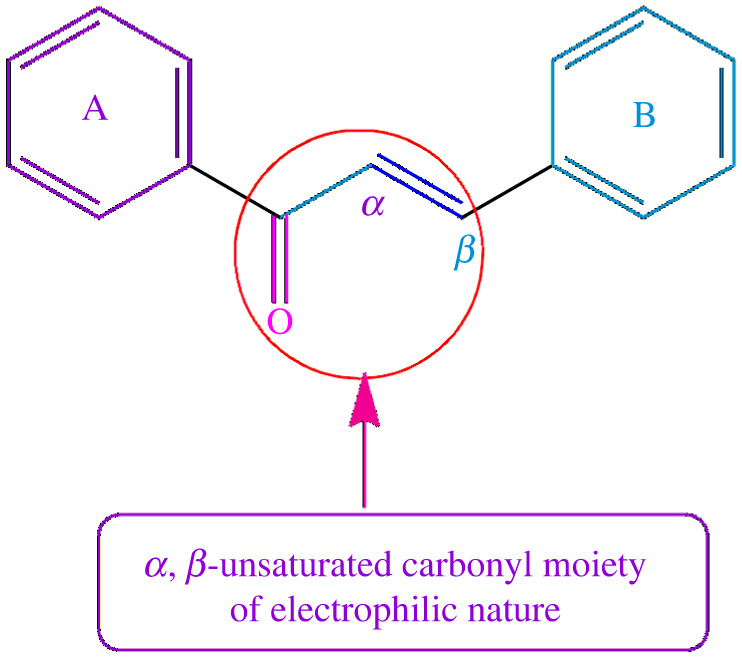
Chemical structure of chalcone.

A chalcone (trans-1, 3-diaryl-2-proper-1-one) represents a simple chemical moiety of numerous natural substances and is extensively found in fruits, vegetables, teas and other vegetation. The word chalcone originates from the Greek term chalcos which means bronze. Chalcones were originally synthesized in the late 1800s [1–6]. Chalcones are *α*, β-unsaturated ketones comprising two phenyl rings (**A** and **B**) possessing a variety of moieties (figure 1).

Chalcone backbone consists of two benzene rings bonded by a system of three aliphatic carbon atoms. Both the rings of chalcone are interlinked by an efficient electrophilic *α*, β-unsaturated carbonyl structure that adopts a linear or virtually two-dimensional assembly. They have conjugated double bonds and a fully delocalized π-electron distribution on the two phenyl rings [7,8]. Chalcones are recognized as fascinating units related to numerous biological activities [9]. Many chalcones are known to integrate with numerous biomolecules and have cytoprotective and regulative effects, moulding them as possibly appropriate candidates for therapeutic interference in several human disorders [10]. The structural adjustments of the aromatic rings in the chalcone scaffold have resulted in a high-scale diversification that has been recognized as beneficial for the discovery of novel therapeutic candidates. Therefore, chalcones have continuously attracted the interest of medicinal chemists both in industry and academia. They are well-recognized for a wide array of bioactivities such as anticancer, antimicrobial, antioxidative, cytotoxic, antiviral and anti-inflammatory, etc. [11]. The growth of drug-resistant microorganisms has produced a need for new antimicrobial drug candidates. Synthetic as well as natural chalcones have exhibited encouraging antimicrobial activities. Chalcones hinder numerous targets responsible for antibiotic resistance growth, excelling the conventional antibiotics. Moreover, chalcones are also known to behave as potential antifungal and antileishmanial agents. Chalcones and their analogues present probable solutions to overcome antimicrobial resistance, leading to worldwide health interventions [12,13]. Although chalcones have several medicinal uses, their comprehensive bioactivity spectrum displays an occasional target profiling, which presents a demanding role in their clinical usage. Owing to the electrophilic character of *α, β*-unsaturated carbonyl scaffold, it can make irreversible links with organic macromolecules, triggering several side effects, for instance, mutagenicity, carcinogenicity and allergic reactions [1,14]. By contrast, the decoration of the aromatic structure with various substituents can efficiently influence the chalcone's reactivity. Therefore, the synthesis of substituted chalcone is particularly important for developing novel drug candidates with improved therapeutic applications [8].

The facile synthesis of chalcones and their potent biological activities encouraged us to synthesize simple chalcone derivatives. Based on a literature survey, base-catalysed Claisen–Schmidt condensation [15] was chosen for the synthesis of 17 chalcone derivatives (**3a**–**3q**) as potential antibacterial, antifungal and antileishmanial agents. It is noteworthy that the synthesis of chalcone (**3q**) is reported for the first time. All chalcones were well characterized and evaluated for their biological activities. The experimental results of this study were further supported by *in silico* investigations.

## 2. Experimental

### 2.1. General

All starting materials were purchased from Sigma-Aldrich (São Paulo, Brazil) and were used without further purification. The progress of chemical reactions was observed via thin-layer chromatography (TLC) utilizing pre-coated silica gel 60 (0.25 mm thickness) plates (Merck, São Paulo, Brazil) with ethyl acetate (Synth, São Paulo, Brazil) and n-hexane (Dinâmica, São Paulo, Brazil) as solvents. TLC plates were investigated under ultraviolet light (*λ* = 254 nm) or developed in iodine vapours (Sigma-Aldrich, São Paulo, Brazil). Melting points were recorded in open capillaries on the Gallenkamp melting point instrument (Sanyo Gallenkamp, Southborough, UK) and are uncorrected. The nuclear magnetic resonance (NMR) spectra were recorded on Bruker AV-200 NMR spectrometer (Bruker BioSpin, Rheinstetten, Germany). Chemical shifts were expressed relative to tetramethylsilane (TMS) (Cambridge Isotope Laboratories Inc. Massachusetts, USA) as a reference using either Chloroform-d or Dimethyl Sulfoxide-D6 as a solvent (Sigma-Aldrich, São Paulo, Brazil). Multiplicities were expressed in a typical manner; s (singlet), d (doublet), dd (doublet of doublet), t (triplet), q (quartet), m (multiplet). The NMR data were provided as follows; chemical shift (*δ*) in ppm, multiplicity, and *J* values in hertz (Hz). The IR spectra were obtained on FT-EQUINOX 55 IR spectrophotometer (Bruker Optik GmbH, Württemberg, Germany) as KBr discs. Mass spectra were documented via Xevo® G2-XS Mass spectrometer (Waters Corporation, Milford, USA).

### 2.2. Synthesis

A single-step, simple methodology was adopted to produce the target chalcones via Claisen–Schmidt condensation. The method involves the condensation of benzaldehydes and acetophenones using a powerful base as a catalyst in a liquid solvent for a few hours. The chalcones are obtained from the aldol product through dehydration involving an enolate pathway. This methodology is widely used due to its experimental leniency and is immensely productive for the generation of the C=C with slight restrictions regarding the intricacy of the molecules. The existence of electron-attracting groups in aldehydes preferred condensation by a base [16,17].

### 2.3. A common methodology for the chalcones synthesis (**3a**–**q**)

**Scheme 1.**
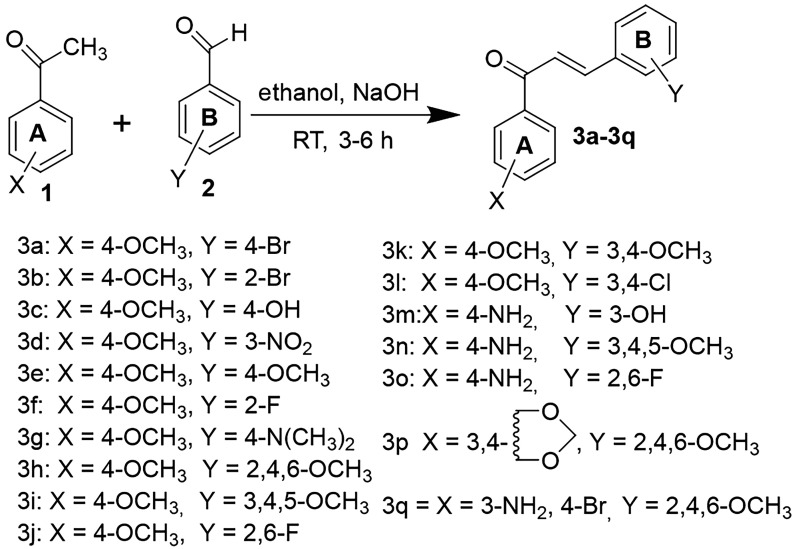
Synthesis of the selected chalcones (**3a**–**q**) via base-catalysed Claisen–Schmidt condensation.

**Scheme 2.**
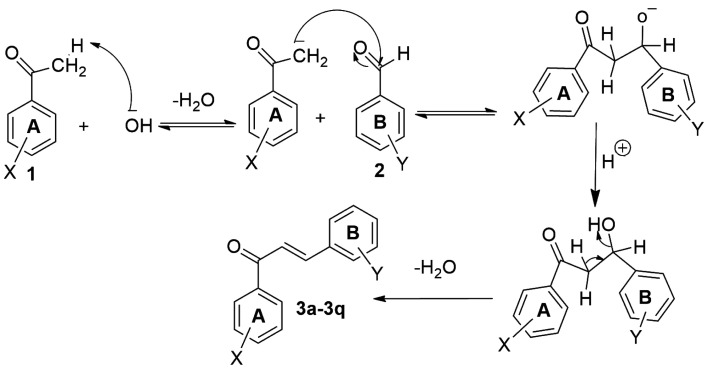
Mechanism for the synthesis of the selected chalcones (**3a**–**q**) via base-catalysed Claisen–Schmidt condensation.

Equal quantities (8 mmol) of acetophenones and respective benzaldehydes were stirred till their dissolution in the least amount of absolute ethanol. Subsequently, 40% aqueous sodium hydroxide solution (15 ml) was added gradually to the mixture followed by continuous stirring for 24 h at room temperature. The formation of the target compounds was confirmed by TLC employing silica gel-G. Upon conclusion of the reaction, the mixture was transferred to a bath of ice cubes followed by its acidification with dilute HCl. The obtained solid was filtered off and dried up. The crude chalcones were purified by recrystallization in absolute ethanol [18–21]. The synthesis and mechanism of the target chalcones (**3a**–**q**) via base-catalysed Claisen–Schmidt condensation are given in schemes 1 and 2, respectively. All the target chalcone derivatives were subsequently characterized by spectroscopic techniques (electronic supplementary material, figures S1–S52).

(E)-3-(4-bromophenyl)-1-(4-methoxyphenyl)prop-2-en-1-one **(3a)**: White crystals; 1.65 g; Yield = 65%; m.p. 138°C, HR-MS (ESI) *m/z*: calculated for C_16_H_13_BrO_2_ [M + H]^+^: 317.01, found: 317.0189; IR (KBr) cm^−1^: 3015 (=C-H_aromatic symmetric stretch_), 2837 (C-H_Symmetric stretch for the O-CH3_), 1661 (-C=O _stretch_), 1604 (C=C _chain_), 1177 (C-O-C _aryl alkyl ether_); ^1^H NMR (200 MHz, CDCl_3_) *δ* 8.08–7.96 (m, 2H), 7.73 (d, *J* = 15.7 Hz, 1H), 7.57 (d, *J* = 3.1 Hz, 1H), 7.53 (d, *J* = 3.4 Hz, 3H), 7.48 (d, *J* = 2.1 Hz, 1H), 7.04–6.91 (m, 2H), 3.90 (s, 3H).

(E)-3-(2-bromophenyl)-1-(4-methoxyphenyl) prop-2-en-1-one **(3b)**: Yellow powder; 1.42 g; Yield = 56%; m.p. 75.5°C; HR-MS (ESI) *m/z*: calculated for C_16_H_13_BrO_2_ [M + H]^+^: 317.01, found: 317.0184; IR (KBr) cm^−1^: 3030 (=C-H_aromatic symmetric stretch_), 2837 (C-H _Symmetric_ _stretch for the O-CH3_), 1652 (-C=O stretch), 1604 (C=C _chain_), 1183 (C-O-C _aryl alkyl ether_); ^1^H NMR (200 MHz, DMSO) *δ* 8.25–8.09 (m, 3H), 7.95 (d, *J* = 1.4 Hz, 2H), 7.72 (dd, *J* = 7.8, 1.5 Hz, 1H), 7.54–7.29 (m, 2H), 7.09 (d, *J* = 8.5 Hz, 2H), 3.86 (s, 3H).

(E)-3-(3-hydroxyphenyl)-1-(4-methoxyphenyl)prop-2-en-1-one **(3c)**: Yellow crystals; 1.4 g; Yield = 69%; m.p. = 163–164°C; HR-MS (ESI) *m/z*: calculated for C_16_H_14_O_3_ [M + H]^+^: 255.10, found: 255.1024; IR (KBr) cm^−1^: 3337 (-O-H stretch.), 2843 (-C-H _Symmetric_ _stretch for the O-CH3_), 1652 (-C=O stretch), 1174 (C-O-C _aryl alkyl ether_); ^1^H NMR (200 MHz, DMSO-d6) *δ* 9.69 (s, 1H), 8.22–8.06 (m, 2H), 7.83 (dd, *J* = 15.7, 2.0 Hz, 1H), 7.60 (dd, *J* = 15.6, 1.9 Hz, 1H), 7.36–7.15 (m, 3H), 7.15–6.95 (m, 2H), 6.93–6.62 (m, 1H), 3.86 (s, 3H).

(E)-1-(4-methoxyphenyl)-3-(3-nitrophenyl)prop-2-en-1-one **(3d)**: White powder; 1.7 g; Yield = 89%; m.p. = 151–152°C; HR-MS (ESI) *m/z*: calculated for C_16_H_13_NO_4_ [M + H]^+^: 284.09, found: 284.0918; IR (KBr) cm^−1^: 2846 (-C-H _Symmetric_ _stretch for the O-CH3_), 1664 (-C=O _stretch_), 1610 (C=C _chain_), 1167 (C-O-C _aryl alkyl ether_), 1521 (N–O _asymmetric_ _stretch_), 1355 (N–O_symmetric stretch_) ^1^H NMR (200 MHz, CDCl_3_) *δ* 8.51 (d, *J* = 2.1 Hz, 1H), 8.25 (dd, *J* = 8.1, 2.2 Hz, 1H), 8.13–8.00 (m, 2H), 7.95–7.85 (m, 1H), 7.79 (s, 1H), 7.69 (s, 1H), 7.61 (t, *J* = 8.0 Hz, 1H), 7.06–6.93 (m, 2H), 3.91 (s, 3H).

(E)-1,3-bis(4-methoxyphenyl)prop-2-en-1-one **(3e)**: Yellowish crystals; 1.4 g; Yield = 65%; m.p. = 103–104°C; HR-MS (ESI) *m/z*: calculated for C_17_H_16_O_3_ [M + H]^+^: 269.11, found: 269.1196; IR (KBr) cm^−1^: 3015 (=C-H_aromatic symmetric stretch_), 2840 (-C-H _Symmetric_ _stretch for the O-CH3_), 1655 (-C=O _stretch_), 1164 (C-O-C _aryl alkyl ether_); ^1^H NMR (200 MHz, CDCl_3_) *δ* 8.09–7.97 (m, 2H), 7.78 (d, *J* = 15.6 Hz, 1H), 7.66–7.56 (m, 2H), 7.43 (d, *J* = 15.6 Hz, 1H), 7.03–6.89 (m, 4H), 3.89 (s, 3H), 3.86 (s, 3H).

(E)-3-(2-fluorophenyl)-1-(4-methoxyphenyl)prop-2-en-1-one **(3f)**: White crystals; 1.7 g; Yield = 83%; m.p. = 105–106°C; HR-MS (ESI) *m/z*: calculated for C_16_H_13_FO_2_ [M + H]^+^: 257.09, found: 257.0995; IR (KBr) cm^−1^: 2846 (-C-H _Symmetric_ _stretch for the O-CH3_), 1655 (-C=O), 1610 (-C=C- _stretch_), 1231 (-C-F _stretch_), 1183 (C-O-C _aryl alkyl ether_); ^1^H NMR (200 MHz, CDCl_3_) *δ* 8.12–8.00 (m, 2H), 7.91 (d, *J* = 15.9 Hz, 1H), 7.74–7.59 (m, 2H), 7.37 (ddd, *J* = 7.3, 5.3, 2.0 Hz, 1H), 7.26–7.07 (m, 2H), 7.00 (dd, *J* = 9.3, 2.4 Hz, 2H), 3.86 (s, 3H).

(E)-3-(4-(dimethylamino)phenyl)-1-(4-methoxyphenyl)prop-2-en-1-one **(3g)**: Yellow powder; 1.4g; Yield = 62%; m.p. = 124–125°C; HR-MS (ESI) *m/z*: calculated for C_18_H_19_NO_2_ [M + H]^+^: 282.14, found: 282.1551; IR (KBr) cm^−1^: 3031 (=C-H_aromatic symmetric stretch_), 1600 (-C=O), 1253 (-C-N stretch), 1164 (C-O-C _aryl alkyl ether_); ^1^H NMR (200 MHz, CDCl_3_) *δ* 8.03 (dd, *J* = 9.4, 2.4 Hz, 2H), 7.79 (d, *J* = 15.4 Hz, 1H), 7.56 (dd, *J* = 10.2, 3.4 Hz, 2H), 7.35 (d, *J* = 15.4 Hz, 1H), 6.98 (dd, *J* = 9.2, 2.4 Hz, 2H), 6.80–6.56 (m, 2H), 3.89 (s, 3H), 3.05 (s, 6H).

(E)-1-(4-methoxyphenyl)-3-(2,4,6-trimethoxyphenyl)prop-2-en-1-one **(3h)**: Yellowish crystals; 1.5 g; Yield = 57%; m.p. = 135–136°C; HR-MS (ESI) *m/z*: calculated for C_19_H_20_O_5_ [M + H]^+^: 329.13, found: 329.1459; IR (KBr) cm^−1^: 3104 and 3000 (=C-H_aromatic symmetric stretch_), 2840 (-C-H _Symmetric_ _stretch for the O-CH3_), 1652 (-C=O _stretch_), 1158 (C-O-C _aryl alkyl ether_); ^1^H NMR (200 MHz, CDCl_3_) *δ* 8.20 (s, 1H), 8.06–7.99 (m, 2H), 7.88 (d, *J* = 15.8 Hz, 1H), 6.97 (dd, *J* = 9.2, 2.4 Hz, 2H), 6.14 (d, *J* = 2.1 Hz, 2H), 3.91 (s, 6H), 3.87 (s, 3H), 3.86 (s, 3H).

(E)-1-(4-methoxyphenyl)-3-(3,4,5-trimethoxyphenyl)prop-2-en-1-one **(3i)**: Yellowish crystals; 1.64 g; Yield = 63%; m.p. = 130–132°C; HR-MS (ESI) *m/z*: calculated for C_19_H_20_O_5_ [M + H]^+^: 329.13, found: 329.1398; IR (KBr) cm^−1^: 3308 and 3002 (=C-H_aromatic symmetric stretch_), 2843 (-C-H _Symmetric_ _stretch for the O-CH3_), 1655 (-C=O _stretch_), 1610 (C=C _chain_), 1183 (C-O-C _aryl alkyl ether_); ^1^H NMR (200 MHz, CDCl_3_) *δ* 8.11–7.99 (m, 2H), 7.73 (d, *J* = 15.5 Hz, 1H), 7.42 (dd, *J* = 15.6, 0.9 Hz, 1H), 7.06–6.94 (m, 2H), 6.90–6.83 (m, 2H), 3.93 (s, 6H), 3.91 (s, 6H).

(E)-3-(2,6-difluorophenyl)-1-(4-methoxyphenyl)prop-2-en-1-one **(3j)**: White powder; 1.6 g; Yield = 73%; m.p. = 88.4–90.5°C; HR-MS (ESI) *m/z*: calculated for C_16_H_12_F_2_O_2_ [M + H]^+^: 275.08, found: 275.0895; IR (KBr) cm^−1^: 3098 and 3053 (=C-H_aromatic symmetric stretch._), 2843 (-C-H _Symmetric_ _stretch for the O-CH3_), 1664 (-C=O _stretch_), 1613 (C=C _chain_), 1167 (C-O-C _aryl alkyl ether_); ^1^H NMR (200 MHz, CDCl_3_) *δ* 8.06 (dd, J = 9.1, 2.4 Hz, 2H), 7.88 (d, J = 2.5 Hz, 2H), 7.34 (d, J = 7.3 Hz, 1H), 6.99 (dd, J = 8.9, 2.3 Hz, 4H), 3.90 (s, 3H).

(E)-3-(3,4-dimethoxyphenyl)-1-(4-methoxyphenyl)prop-2-en-1-one **(3k)**: Pale yellow crystals; 1.55 g; Yield = 65%; m.p.= 105–106.4°C; HR-MS (ESI) *m/z*: calculated for C_18_H_18_O_4_ [M + H]^+^: 299.12, found: 299.1310; IR (KBr) cm^−1^: 3079 and 3053 (=C-H_aromatic symmetric stretch._), 2843 (-C-H _Symmetric_ _stretch for the O-CH3_), 1655 (-C=O _stretch_), 1591 (C = C chain); ^1^H NMR (200 MHz, CDCl_3_) *δ* 8.10–7.96 (m, 2H), 7.76 (d, *J* = 15.6 Hz, 1H), 7.40 (d, *J* = 15.6 Hz, 1H), 7.24–7.09 (m, 2H), 7.04–6.94 (m,2H), 6.90 (d, *J* = 8.3 Hz, 1H), 3.96 (s, 3H), 3.93 (s, 3H), 3.89 (s, 3H).

(E)-3-(3,4-dichlorophenyl)-1-(4-methoxyphenyl)prop-2-en-1-one **(3** **l)**: White crystals; 1.5 g; yield = 61%; m.p. = 185–186°C; HR-MS (ESI) *m/z*: calculated for C_16_H_12_Cl_2_O_2_ [M + H]^+^: 307.02, found: 307.0294; IR (KBr) cm^−1^: 3060 and 3018 (=C-H _aromatic symmetric stretch._), 2967 (-C-H _Asymmetric stretch for the O-CH3_), 2837 (-C-H _Asymmetric stretch for the O-CH3_), 1655 (-C=O _stretch_), 1585 (C=C _chain_), 1257 (C-O _Stretch_.); ^1^H NMR (200 MHz, CDCl_3_) *δ* 8.11–7.96 (m, 2H), 7.72 (d, J = 1.6 Hz,1H), 7.65 (s, 1H), 7.55 (s, 1H), 7.52–7.39 (m, 2H), 7.05–6.94 (m, 2H), 3.90 (s,3H).

(E)-1-(4-aminophenyl)-3-(3-hydroxyphenyl)prop-2-en-1-one **(3m)**: Yellow powder; 1.2 g; Yield = 63%; m.p. = 142–143°C; HR-MS (ESI) *m/z*: calculated for C_15_H_13_NO_2_ [M + H]^+^: 240.10, found: 240.1041; IR (KBr) cm^−1^ **:** 3423 and 3343 (-N-H _stretch_), 32041 (-O-H stretch), 1642 (-C=O _stretch_), (1591 (C=C _aromatic_), 1180 (-C-O _stretch_); ^1^H NMR (200 MHz, DMSO-*d*_6_) *δ* 9.62 (s, 1H), 7.89 (dd, *J* = 8.0, 2.0 Hz, 2H), 7.81–7.60 (m, 1H), 7.59–7.34 (m, 1H), 7.33–7.11 (m, 3H), 6.82 (d, *J* = 4.4 Hz, 1H), 6.61 (dd, *J* = 8.2, 2.2 Hz, 2H), 6.13 (s, 2H).

(E)-1-(4-aminophenyl)-3-(3,4,5-trimethoxyphenyl)prop-2-en-1-one **(3n)**: Yellow crystals; 1.6 g; Yield = 64%; m.p. = 157–158°C; HR-MS (ESI) *m/z*: calculated for C_18_H_19_NO_4_ [M + H]^+^: 314.13, found: 314.1450; IR (KBr) cm^−1^ **:** 3445 and 3321 (-NH_2_), 3057 and 3009 (=C-H_aromatic symmetric stretch_), 2840 (-C-H _Symmetric_ _stretch for the O-CH3_), 1636 (-C = O _stretch_), 1585 and 1591 (C=C aromatic), 1177 (C=C chain); ^1^H NMR (200 MHz, CDCl_3_) *δ* 8.00–7.86 (m, 2H), 7.70 (d, *J* = 15.5 Hz, 1H), 7.41 (d, *J* = 15.6 Hz, 1H), 6.85 (s, 2H), 6.77–6.64 (m, 2H), 4.16 (s, 2H), 3.92 (s, 6H), 3.89 (s, 3H).

(E)*-*1-(4-aminophenyl)-3-(2,6-difluorophenyl)prop-2-en-1-one **(3o):** Yellow powder; 1.43 g; Yield = 69%; m.p. = 138–139°C; HR-MS (ESI) *m/z*: calculated for C_15_H_11_F_2_NO [M + H]^+^: 260.08, found: 260.0949; IR (KBr) cm^−1^: 3420 and 3337 (-N-H stretch), 3057 (=C-H_aromatic symmetric stretch_), 1655 (-C = O _stretch_), 1633 (-N-H _bend_), 1292 (-C-N _stretch_); ^1^H NMR (200 MHz, CDCl_3_) *δ* 7.99–7.89 (m, 2H), 7.85 (s, 2H), 7.40–7.28 (m, 1H), 6.95 (t, *J* = 8.5 Hz, 2H), 6.72 (d, *J* = 2.1 Hz, 1H), 6.68 (d, *J* = 1.9 Hz, 1H), 4.19 (s, 2H).

(E)-1-(1,3-benzodioxol-5-yl)-3-(2,4,6-trimethoxyphenyl)prop-2-en-1-one **(3p)**: Pale yellow powder; 1.45 g; Yield = 53%; m.p. = 141–142°C; HR-MS (ESI) *m/z*: calculated for C_19_H_18_O_6_ [M + H]^+^: 343.11, found: 343.1203; IR (KBr) cm^−1^: 3111 and 3076 (=C-H_aromatic symmetric stretch_), 2843 (-C-H _Symmetric_ _stretch for the O-CH3_),1655 (-C=O _stretch_), 1566 (C=C _aromatic_), 1116 (C–O–C);^1^H NMR (200 MHz, CDCl_3_) *δ* 8.23 (d, *J* = 15.8 Hz, 1H), 7.83 (d, *J* = 15.8 Hz, 1H), 7.63 (dd, *J* = 8.1, 1.7 Hz, 1H), 7.53 (d, *J* = 1.7 Hz, 1H), 6.87 (d, *J* = 8.1 Hz, 1H), 6.14 (s, 2H), 6.04 (s, 2H), 3.90 (s, 6H), 3.86 (s, 3H).

(E)-1-(3-amino-4-bromophenyl)-3-(2,4,6-trimethoxyphenyl)prop-2-en-1-one (**3q**): Yellow crystals; 2.3 g; yield = 75%; m.p. = 135–136°C; HR-MS (ESI) *m/z*: calculated for C_18_H_18_BrNO_4_ [M + H]^+^: 392.05, found: 392.0529; IR (KBr) cm^−1^: 3452 and 3356 (-N-H_stretch_), 3000 and 2939 (-C-H_aromatic symmetric stretch_), 2837 (-C-H _Symmetric_ _stretch for the O-CH3_),1645 (-C=O _stretch_), 1578 and 1464 (C=C _aromatic stretch_), 1416 (–C-H _bend_), 1034 (C-O _stretch_); ^1^H NMR (200 MHz, CDCl_3_) *δ* 8.23 (d, *J* = 15.9 Hz, 1H), 7.76 (d, *J* = 15.9 Hz, 2H), 7.50 (d, *J* = 8.2 Hz, 1H), 7.43–7.36 (m, 1H), 7.29–7.14 (m, 2H), 6.13 (s, 2H), 3.89 (s, 6H), 3.85 (s, 3H). ^13^C NMR (50 MHz, CDCl_3_) *δ* 190.97, 162.73, 161.27, 143.75, 139.09, 135.78, 131.94, 121.31, 118.78, 114.76, 112.92, 106.07, 90.07, 89.84, 76.52, 75.89, 55.34, 54.92.

## 3. Biological activities

### 3.1. Materials

Three bacterial strains, *Staphylococcus aureus* [ATCC 23235], *Escherichia coli* [ATCC 25922] *Pseudomonas aeruginosa* [ATCC 27853], a fungal strain, *Candida albicans* [ATCC 18804], and a leishmanial strain, *Leishmania major* [ATCC 30012] were all obtained from American Type Culture Collection (ATCC), São Paulo, Brazil.

### 3.2. Antibacterial analysis

Agar well diffusion standard analysis was used to find out the bactericidal activities of the target chalcones (**3a**–**q**). In the present study, one Gram-positive (*E. coli*), and two Gram-negative (*S. aureus* and *P. aeruginosa*) bacterial strains were used for antibacterial assay. Initially, a nutrient broth culture was organized by running the nutrient broth (0.4 g) in distilled water (50 ml), the pH was attuned at 7.0 followed by autoclave [22,23]. Then nutrient agar culture was arranged by the dissolution of nutrient agar medium (2.3 g) in distilled water (100 ml); pH was calibrated at 7.0 followed by autoclave at 121°C. Subsequently, the medium was decanted in Petri plates. Then, 1.175% barium chloride dihydrate (0.05 ml) was stirred with 1% sulfuric acid (9.95 ml) to prepare a 0.5 McFarland standard. A 0.5 McFarland standard conforms nearly to 1.5 × 10^8^ cells ml^−1^. Bacteria were cultured a day before the analysis in nutrient broth suspension. The bacterial inoculants were tuned up to 0.5 McFarland standard followed by their addition to nutrient agar plates. Subsequently, wells (4 mm) were created in the Petri plates. The trial chalcones of three distinct concentrations (1000, 500 and 250 μg ml^−1^) in DMSO were added to the wells of the Petri plates and nurtured at 37°C for 24 h. The inhibitory regions of the trial chalcones were distinguished after 24 h. The whole data were noted carefully. A broth macrodilution assay was utilized to assess the minimum inhibitory concentrations (MICs) of the trial chalcones versus the three bacterial strains. For the correct calculation of MICs, Mueller–Hinton broth (MHB) was chosen as a medium. Small disinfected tubes (1.00 ml) were utilized to carry out this analysis. Separate tubes were employed to prepare various concentrations of the trial chalcones. MHB (500 µl) was dispersed into every glass tube. Afterwards, the three bacterial species and homogeneous bacterial suspension of 0.5 McFarland turbidity were added to the tubes having distinct concentrations of the trial chalcones. A bacterium in an antibiotic-free medium and bacteria with antibiotics were also organized. The tubes were then nurtured for 24 h at 37°C. The inhibitory effect of the trial chalcones was calculated with the maximum dilution without any turbidity. The tube with MIC and without any bacterial proliferation was further diluted to calculate the optimal MICs of the trial chalcone [24].

### 3.3. Antifungal assay

The agar well diffusion technique was applied to measure the antifungal activity of chalcones (**3a**–**q**) versus clinical isolate of *C. albicans* compared with a positive control (chloramphenicol) [22,23]. Initially, Sabouraud dextrose agar (SDA) was organized following the manufacturer's guidelines. Subsequently, the medium was allowed to autoclave at 121°C. The medium was then transferred into petri plates. Then, 1.175% barium chloride dihydrate (0.05 ml) was shaken with 1% sulfuric acid (9.95 ml) to prepare a 0.5 McFarland standard. The desired inoculant was organized by consuming the *C. albicans* from a 24 h culture on Sabouraud dextrose agar. The cell suspension was prepared in a pasteurized saline medium (0.85%). The turbidity of the fungal cell suspension was attuned by a spectrophotometer at 530 nm to get an ultimate concentration corresponding to that of a 0.5 McFarland standard. The SDA plates from 0.5 McFarland standard inoculant were employed to prepare the fungal cell lawn. Subsequently, wells (4 mm) were organized in the Petri plates. The trial chalcones of various concentrations (500, 250 and 100 µg ml^−1^) were added to the wells of the Petri plates followed by their incubation at 35°C for 48 h. The zones of inhibition of the trial chalcones were determined after 48 h with chloramphenicol as a positive control. The data were noted and subsequently elucidated.

### 3.4. Antileishmanial assay

Antileishmanial assay of the chalcones was performed using formerly reported procedures of Habtemariam with slight modification [25]. Promastigotes culture of *L. major* was cultivated in RPMI 1640 medium and nurtured at 24°C for 6–7 days. To make the stock solution of the selected chalcones for antileishmanial evaluation, each chalcone derivative (1 mg) was dissolved in DMSO (1 ml) to produce a concentration of 1000 µg ml^−1^. Successive dilution of the stock solution was then carried out. The 96-well microtitre plate wells were filled approximately with 180 µl of 199 media. For every trial chalcone, 20 µl was added to the first well followed by serial dilution to retain an ultimate volume of 180 µl. Then, 20 µl was disposed of from the final well. Around, 100 µl of the parasite was added to every well and two rows were chosen for positive and negative control. DMSO and Amphotericin B were chosen as negative and positive controls respectively and both were sequentially diluted in the RPMI 1640 medium. Microtitre plates were hatched in a shaker incubator at 24°C for 72 h. The analysis was carried out in triplicate. At the end of the incubation period, 20 µl was extracted from every dilution and placed on an upgraded Neubauer counting chamber, and living parasites were calculated underneath a microscope. Percentage inhibition was determined for each compound and the IC_50_ values of trial chalcones having antileishmanial potential were computed by Graph Prism software.

## 4. *In* *silico* study

### 4.1. Molecular docking analysis

Molecular docking is a computational tool employed to elucidate complex structures formed by the interaction of two or more molecules. Its primary objective is to predict the three-dimensional conformation of a ligand inside the binding pocket of the selected receptor protein. Molecular docking makes a significant contribution to drug discovery. The technique has rapidly attained a valued status in the contemporary set-up of drug design [26]. For our docking study, we select proteins from the different bacterial strains. AutoDock Vina [27] integrated within PyRx version 0.8 (https://pyrx.sourceforge.io) [28] was employed to perform the molecular docking. Initially, ChemDraw software was used to construct chalcones **3q** and **3i**. They were then uploaded into PyRx using the built-in OpenBabel graphical user interface (http://openbabel.org). The energy of the two ligands was minimized using the universal force field (UFF) with overall steps set to 200. Afterward, all ligands were converted into .pdbqt format. The three-dimensional crystallographic structures of five proteins, namely carotenoid dehydrosqualene synthase (PDB ID: **3W7F**) of *S. aureus* [29,30], DNA gyrase B (PDB ID: **4WUB**) of *E. coli* [31], pseudomonas quinolone signal response protein (PDB ID: **2Q0J**) of *P. aeruginosa* [32], leishmanolysin (PDB ID: **1LML**) of *L. major* [33,34] and Secreted aspartic proteinase 5 (PDB ID: **2QZX**) of *C. albicans* [35], were acquired from the Protein Data Bank (https://www.rcbs.org) in PDB format. Initially, water molecules and heteroatoms were eliminated, and then missing atoms were connected to residues. Subsequently, polar hydrogen atoms and Kollman charges were added. The selected proteins were then loaded to PyRx and changed to pdbqt format. During the processing, the exhaustiveness level was set to 24 to ensure a comprehensive search. The Computed Atlas of Surface Topography of proteins (CASTp) server was employed to predict the active site pockets [36]. The Poc ID with a greater volume and surface area was selected as an active pocket. A grid box was positioned covering the amino acid residuals comprising the active spot pocket. The results were then analysed using the Discovery Studio visualizer.

### 4.2. Density functional theory analysis

Density functional theory (DFT) represents a quantum mechanical technique that attained much consideration and was extensively applied to the drug discovery process in the recent past. Moreover, DFT is cost-effective and explains molecular and biological systems with high accuracy. Nowadays, it is regarded as one of the most efficient techniques for therapeutic investigations [37]. In the DFT study, the three-dimensional structures of selected chalcone derivatives were optimized using the G09 software [38]. Subsequently, harmonic vibrational frequencies were determined at the equivalent theoretical extent to confirm the ground state stability of the optimized structures. A combination of the Lee–Yang–Parr (B3LYP) technique and Becke's three-parameter hybrid model was employed to determine the single-point energy calculations [39,40]. To get a better understanding of the chemical reactivity of selected chalcones, frontier molecular orbital (FMO) analysis was performed.

## 5. Results and discussion

**Figure 2.**
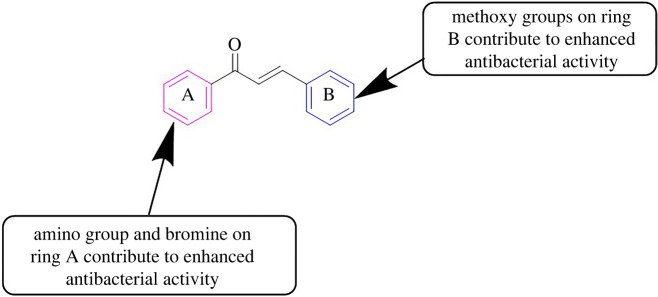
SAR results evaluated in this work, using amino and methoxy substituents.

Antimicrobial resistance (AMR) happens when microorganisms like viruses, fungi, bacteria and parasites become adapted and reproduce in the presence of medicines that were utilized to hinder them in the past [41,42]. AMR is viewed as a principal threat to community health units worldwide. Though several steps were taken in the recent past to resolve the problem, AMR still exists as a serious health risk worldwide. It is accredited to the wrong and excessive use of antimicrobial drugs in the healthcare units as well as the agricultural sector. Additionally, the amendment in the DNA order, inherent evolution and transference of the impervious genes through horizontal gene transport of bacterial cells are the noteworthy aspects responsible for AMR. Bearing in mind the genuine risk of AMR, the antimicrobial assays of the synthesized chalcones versus the chosen species of bacteria, fungi and leishmania were carried out [12,43]. A literature survey indicates that chalcone derivatives act as effective antimicrobial agents [44–46]. In the present study, the antibacterial potency of the chalcones (**3a**–**q**) was screened versus three bacterial strains i.e. *E. coli, S. aureus* and *P. aeruginosa*. The zone of inhibition was chosen to correlate the antibacterial potential with that of chloramphenicol (standard drug). The results of the antibacterial assay (table 1) showed that some of the chalcones displayed good to excellent activities against the tested species. Among the chalcone series, methoxychalcones (**3e, 3h, 3i, 3k, 3n, 3p** and **3q**) and halosubstituted chalcones (**3j, 3l,** and **3o**) showed significant antibacterial activities against the three bacterial strains with reference to the standard drug. Trimethoxychalcones **3p** and **3q** (at 1000 µg ml^−1^ concentration) were observed to be the most potent antibacterial agents. The zone of inhibition values for compound **3p** (at 1000 µg ml^−1^ concentration) were noticed to be 32, 30 and 28 mm versus *E. coli, S. aureus* and *P. aeruginosa,* respectively. Similarly, the compound **3q** (at 1000 µg ml^−1^ concentration) showed antibacterial potential with its zone of inhibition values of 33, 30 and 34 mm versus *E. coli, S. aureus* and *P. aeruginosa,* respectively. A decrease in the antibacterial activity was noted with the decrease in the concentration of chalcone derivatives. The existence of the amino moiety at position 3 in ring A and three methoxy moieties in ring B led to the strongest antibacterial effect of compound **3q**. Structure-activity relationship (SAR) studies reveal that methoxychalcones exhibited better antibacterial effects compared with other chalcones. Therefore, the incorporation of amino groups in ring A and methoxy moieties in ring B of the chalcone scaffold can lead to enhanced antibacterial potential (figure 2). The overall results of the antibacterial assay for compounds (**3a-3q**) are stated in table 1.

**Table 1.** Antibacterial potential of the chalcones (**3a**–**q**) at three distinct concentrations versus different bacteria*.* The zone of inhibition is given in mm. Zone of inhibition diameter in mm. (Activity): above 18 mm (significant activity), 16–18 mm (good activity) 13–15 mm (low activity), 9–12 mm (non-significant), and below 9 mm (no activity).

s. no.	code	*E. coli* [ATCC 25922]			*S. aureus* [ATCC 23235]			*P. aeruginosa* [ATCC 27853]		
		1000 μg ml^−1^	500 μg ml^−1^	250 μg ml^−1^	1000 μg ml^−1^	500 μg ml^−1^	250 μg ml^−1^	1000 μg ml^−1^	500 μg ml^−1^	250 μg ml^−1^
1	**3a**	11	06	—	15	08	03	18	10	—
2	**3b**	10	05	—	14	7	–	17	09	—
3	**3c**	17	11	09	19	08	06	21	13	07
4	**3d**	19	15	12	20	12	10	18	14	11
5	**3e**	19	12	13	21	14	11	22	15	06
6	**3f**	18	11	09	21	11	10	14	11	13
7	**3g**	25	20	18	26	18	16	23	19	16
8	**3h**	28	21	17	26	17	13	29	17	14
9	**3i**	29	17	16	28	23	19	30	17	14
10	**3j**	20	21	18	30	26	20	25	20	16
11	**3k**	22	19	15	23	22	18	24	20	16
12	**3l**	26	10	8	23	16	11	24	8	05
13	**3m**	18	12	11	14	11	09	16	11	07
14	**3n**	30	22	16	29	13	11	31	12	08
15	**3o**	27	12	11	22	13	06	26	11	10
16	**3p**	32	22	09	30	12	10	28	13	11
17	**3q**	33	14	12	30	12	03	34	15	09
18	chloramphenicol	38	35	25	38	33	26	35	24	22

The target chalcones (**3a**–**q**) were also screened *in vitro* for their antifungal effects at various concentrations versus *C. albicans* (table 2). Most of the tested chalcones showed inhibitory potential versus *C. albicans*. It is obvious from the data that the antifungal activity of the tested chalcones increased with the increase in the concentrations of the chalcones. The data further revealed that compounds **3c, 3d**, **3e, 3f, 3j** and **3m** exhibited good antifungal activity with their zone of inhibitions noted to be 17, 16, 17, 18, 18 and 16 mm, respectively at 500 µg ml^−1^ as compared with fluconazole (standard drug). Chalcones **3 g, 3 h, 3i, 3k, 3 l, 3n, 3o, 3p** and **3q** (all of 500 µg ml^−1^ concentration) showed significant antifungal activity with their zone of inhibitions recorded to be 20, 28, 29, 19, 22, 21, 22, 24 and 25 mm, respectively. Chalcone **3i** (500 µg ml^−1^) was noted to be the most potent among all the synthesized chalcones with its zone of inhibition being 29 mm. SAR investigation reveals that increasing the number of methoxy moieties on benzene rings (A and B) leads to improvement in the antifungal effect of the chalcones. The overall results of the antifungal analysis for compounds (**3**–**3q**) are stated in table 2.

**Table 2.** Antifungal potential of chalcones (**3a**–**q**) at three different concentrations versus *C. albicans.* The zone of inhibition is shown in mm. Zone of inhibition diameter in mm. (Activity): above 18 mm (significant activity), 16–18 mm (good activity) 13–15 mm (low activity), 9–12 mm (non-significant), and below 9 mm (no activity).

s. no.	code	zone of inhibition (in mm) versus *C. albicans* [ATCC 18804]		
		500 μg ml^−1^	250 μg ml^−1^	100 μg ml^−1^
1	**3a**	12	08	06
2	**3b**	11	06	04
3	**3c**	17	11	04
4	**3d**	16	11	09
5	**3e**	17	14	11
6	**3f**	18	12	14
7	**3g**	20	16	10
8	**3h**	28	18	12
9	**3i**	29	20	14
10	**3j**	18	16	14
11	**3k**	19	13	10
12	**3l**	22	18	12
13	**3m**	16	13	10
14	**3n**	21	15	11
15	**3o**	22	14	16
16	**3p**	24	18	11
17	**3q**	25	20	13
18	fluconazole	32	28	20

**Figure 3.**
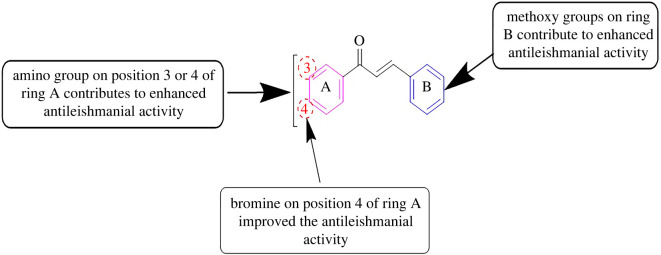
SAR results evaluated in this work, using amino, bromo and methoxy substituents.

The synthesized chalcones (**3a**–**q**) were also investigated for their antileishmanial activities against the leishmania parasite in terms of their half-maximal inhibitory concentration (IC_50_) values (table 3). The data revealed that chalcones **3e, 3f, 3g, 3h, 3i, 3l** and **3p** showed good antileishmanial potential. Chalcones **3n** and **3q** exhibited significant potency with their IC_50_ values noted to be 0.59 ± 0.20 and 0.59 ± 0.12 µg ml^−1^, respectively with reference to Amphotericin B (standard drug). SAR analysis showed that the existence of amino groups on ring A and methoxy moieties on ring B (**3n** and **3q**) caused a significant antileishmanial effect of the chalcones. Furthermore, the incorporation of bromine on aromatic ring A can also lead to improvement in the antileishmanial activity of chalcones (figure 3). The overall results of the antileishmanial assay for compounds (**3a**–**q**) are stated in table 3.

**Table 3.** IC_50_ values of the chalcones (**3a**–**q**) against *L. major* [ATCC 30012]. s.e.m. = standard error mean (experiment run in triplicate). Criteria for IC_50_ are: significant activity (0.56–0.59), good activity (0.60–0.69, moderate activity (0.70–0.79), low activity (0.80–0.95), non-significant activity (0.99).

s. no.	codes	IC_50_ ± s.e.m. (μg ml^−1^)
1	**3a**	0.84 ± 0.10
2	**3b**	0.82 ± 0.09
3	**3c**	1.37 ± 0.07
4	**3d**	0.79 ± 0.16
5	**3e**	0.69 ± 0.08
6	**3f**	0.63 ± 0.12
7	**3g**	0.63 ± 0.12
8	**3h**	0.64 ± 0.10
9	**3i**	0.63 ± 0.09
10	**3j**	0.97 ± 0.07
11	**3k**	0.88 ± 0.16
12	**3l**	0.60 ± 0.08
13	**3m**	0.78 ± 0.12
14	**3n**	0.59 ± 0.20
15	**3o**	0.86 ± 0.08
16	**3p**	0.61 ± 0.12
17	**3q**	0.59 ± 0.12
18	standard (Ampotericine B)	0.58 ± 0.20

In addition, *in silico* analyses were performed to aid the experimental results which are described in detail below.

**Figure 4.**
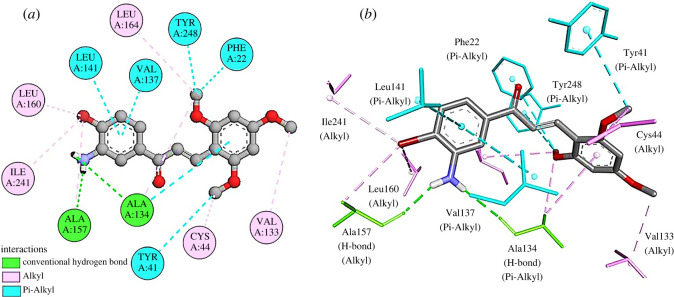
(*a*) Two-dimensional and (*b*) three-dimensional interactions of **3W7F-3q**.

For molecular docking of dehydrosqualene synthase, the active site was determined using the CASTp server, and the Poc ID 1 with a volume of 639.672 Å^3^ and surface area (SA) of 688.769 Å^2^ was selected as an active site. Compound **3q** exhibited a binding energy of −7.7 kcal mol^−1^ with CrtM and formed 15 interactions including two H-bonds with residues Ala157 and Ala134, six pi-alkyl bonds with residues Tyr41, Ala134, Phe22, Tyr248, Val137 and Leu141. Additionally, seven alkyl bonds with residues Val133, Cys44, Ile241, Leu160, Ala157, Ala134 and Leu164 were also established (figure 4 *a,b*). By forming such interactions in the vicinity of the active site, compound **3q** can have the ability to hinder the normal function of the CrtM protein of *S. aureus* [47].

**Figure 5.**
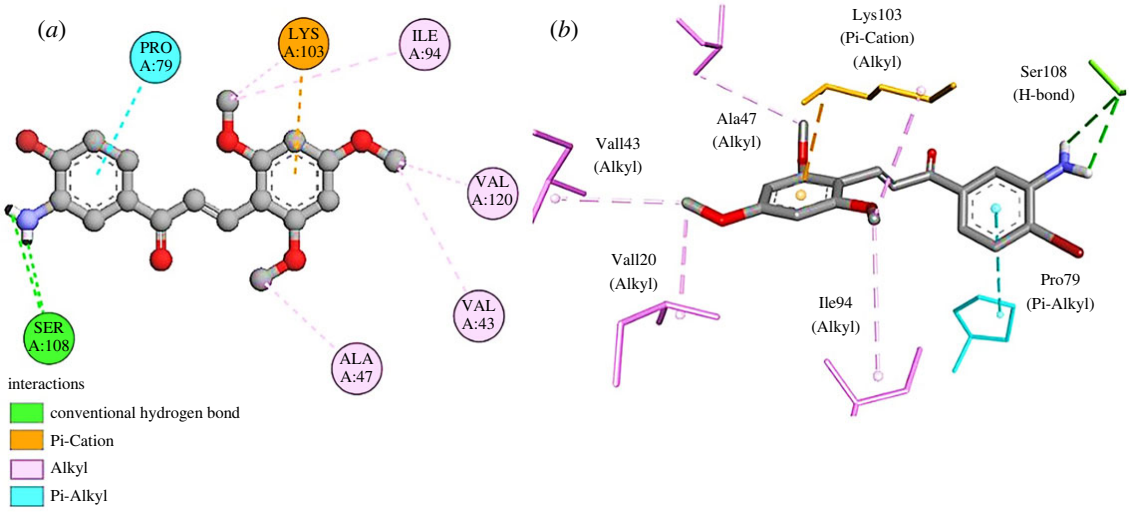
(*a*) Two-dimensional and (*b*) three-dimensional interactions of **4WUB-3q**.

Similarly, Pocket ID 1 with a volume of 151.668 Å^3^ and SA of 287.309 Å^2^ was chosen as an active pocket of DNA gyrase B. The compound **3q** displayed a binding energy of −7.9 kcal mol^−1^^ ^with DNA gyrase B, forming a total of nine interactions. These interactions comprised two H-bonds generated by residue Ser108. The residues Ile94, Val120, Val43, Ala47 and Lys103 formed alkyl bonds with compound **3q**. Moreover**,** pi-alkyl and pi-cation bonds were established by residues Pro79 and Lys103, respectively (figure 5 *a,b*). Targeting the ATPase site can be a useful strategy in inhibiting the DNA gyrase activity. There are sites 1, 2, and Mg-ATP binding spots in the ATPase site of the B domain of DNA gyrase [48]. Compound **3q** is forming bonds with residues Ile94 and Val120 from site 1, residues Lys103 and Ser108 from site 2, and residues Ala47 and Val43 from the Mg-ATP binding site, thus inhibiting the ATPase binding site, leading to the suppression of GyrB activity.

**Figure 6.**
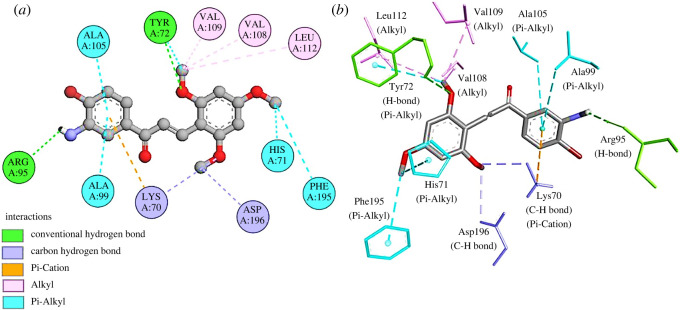
(*a*) Two-dimensional and (*b*) three-dimensional interactions of **2Q0J-3q**.

The active site with pocket ID 1 of volume of 85.306 Å^3^ and SA of 114.140 Å^2^ of pseudomonas quinolone signal response protein (PqsE) of *P. aeruginosa* was selected for docking study. Compound **3q** exhibited a binding energy of −7.1 kcal mol^−1^ with PqsE protein. Compound **3q** formed 13 interactions with the target protein. These interactions include two H-bonds with residues Tyr72, Arg95, and two C-H bonds with residues Lys70, and Asp196. In addition, one pi-cation interaction with residue Lys70, five pi-alkyl bonds with residues Phe195, His71, Ala99, Ala105, Tyr72 and three alkyl bonds with residues Val109, Val108, Leu112 were observed **(** figure 6 *a,b*). The overall fold of PqsE resembles characteristic metallo-β-lactamase hallmarked by an *αβ*/*αβ* sandwich main structure. Compared with other enzymes, PqsE holds two extra R-helices at its C-terminus that encompass the active epicentre. These two helices restrict availability to the active spot and could be able to move as lids that open and close for substrate attachment. Additionally, PqsE attaches to two iron atoms in its active epicentre. These iron atoms remain attached to the active epicentre residues [49]. Compound **3q** interacted with active site residues Tyr72, Lys70 and His71, which are near to the active epicentre of the PqsE protein.

**Figure 7.**
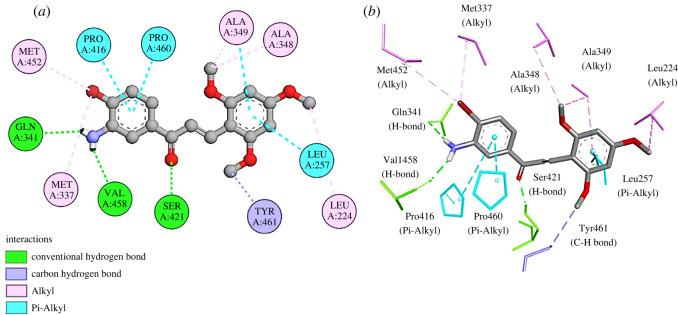
(*a*) Two-dimensional and (*b*) three-dimensional interactions of **1LML-3q**.

Likewise, the active site of leishmanolysin (GP63) was determined through the CASTp server. The pocket ID of 1 with a volume of 618.705 Å^3^ and SA of 686.710 Å^2^ was selected as an active pocket. Compound **3q** showed a binding energy of −7.4 kcal mol^−1^ and established 13 interactions with the target protein. Specifically, it established three H-bonds with residues Gln341, Val458 and Ser421. In addition, it formed five alkyl bonds with residues Leu224, Met337, Met452, Ala349 and Ala348. One carbon-hydrogen bond with residue Tyr461 and four pi-alkyl bonds with residues Pro416, Pro460, Leu257 and Ala349 were noted (figure 7 *a,b*). Leishmanolysin is a tightly packed molecule covering predominantly *β* sheet secondary structure. The protein contains three domains: the N-terminal, central and C-terminal domains. The active spot is contained in the N-terminal domain [50]. Compound **3q** established potential interaction with the key residues in the active spot of GP63 protein. These include Met337, Ala349, Gln341 and Leu257.

**Figure 8.**
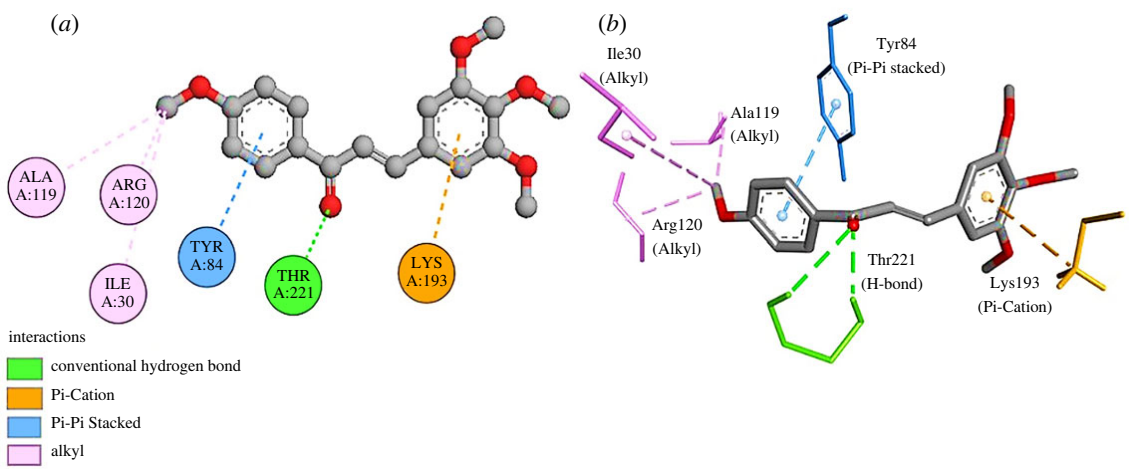
(*a*) Two-dimensional and (*b*) three-dimensional interactions of **2QZX-3i**.

For the molecular docking study of secreted aspartic proteinase (Sap5) with compound **3i**, the active site was determined by the CASTp server. The Pocket ID of 1 with a volume of 618.705 Å^3^ and a SA of 686.710 Å^2^ was chosen as an active pocket. The docking study showed a binding energy of −7.3 kcal mol^−1^ for compound **3i** with sap5. Compound **3i** formed six interactions with the target protein, one H-bond with residue Thr221, three alkyl bondings with residues Ala119, Arg120 and Ile30, one pi-pi stacking interaction with residue Tyr84 and one pi-cation link with residue Lys193 (figure 8 *a,b*). Residues in sap5 form different substrate binding pockets [51]. Compound **3i** formed interactions with residues Ala119, Ile30, and Tyr84 of the S1 substrate binding pocket. Similarly, compound **3i** also established interactions with residues Thr221 and Arg120 of S2, and S3 binding pockets respectively, and possibly resulted in the inhibition of their activity.

The calculated binding energies of the most potent chalcones (**3i** and **3q**) after their molecular docking with the proteins of the selected bacterial, fungal and leishmanial strains are provided (table 4).

**Table 4.** Summary of the docking results of chalcones (**3i** and **3q**).

s. no.	organisms	proteins	chalcone derivatives	binding energy (kcal mol ^−1^)
1	*P. aeruginosa*	PqsE (PDB ID: 2Q0J)	**3q**	−7.1
2	*E. coli*	GyrB (PDB ID: 4WUB)	**3q**	−7.9
3	*S. aureus*	CrtM (PDB ID: 3W7F)	**3q**	−7.7
4	*L. major*	GP63 (PDB ID: 1LML)	**3q**	−7.4
5	*C. albicans*	Sap5 (PDB ID: 2QZX)	**3i**	−7.3

Furthermore, DFT calculations of top-ranked compounds **3i** and **3q** were performed to support experimental and docking results.

**Figure 9.**
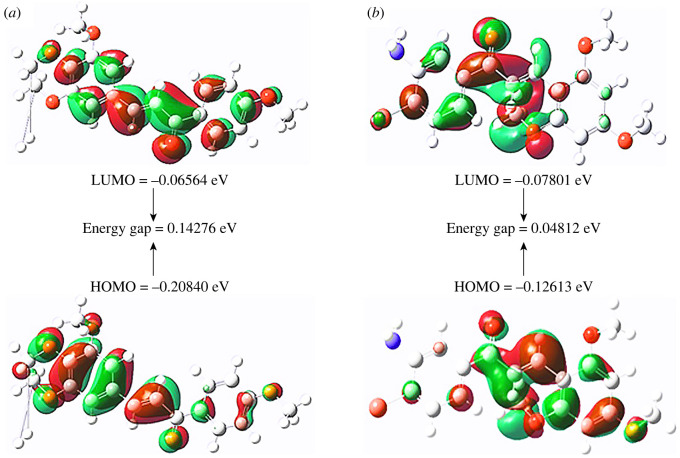
HOMO, LUMO energies, and HOMO-LUMO energy gap (H-L gap) of chalcones (*a*) **3i** and (*b*) **3q**.

FMOs help to determine the chemical reactivity of a molecule by exploring its electron-releasing and electron-withdrawing abilities [52,53]. HOMO–LUMO energy gap calculated from FMOs analysis provides key information regarding the measurement of intermolecular charge transfer and chemical reactivity of molecules; it has been efficiently used for predicting biological activities [54,55]. In general, a smaller energy gap means more chemical reactivity due to the ease with which an electron can be promoted from HOMO to LUMO. On the other hand, a larger energy gap for HOMO-LUMO represents less chemical reactivity [56,57]. The visualization of FMOs along with the corresponding H-L energy gap are shown (figure 9; electronic supplementary material, figures S53 and S54). Energy gaps of 0.14276 eV and 0.04812 eV were reported for **3i** and **3q** compounds, respectively. A low H-L energy gap indicates high reactivity of both compounds (**3i** and **3q)** inferring their high biological impacts.

The dipole moment is another key criterion that affects the biological activities of organic compounds. Studies have been performed to investigate a relationship between the dipole moment and antimicrobial activities of chalcones. The dipole moment of chalcones has been computed in gas as well as solvent phases. The data suggested that chalcone derivatives with low dipole moments exhibited enhanced antibacterial and antifungal activities [58]. In the current study, the DFT optimization of compounds **3i** and **3q** also confirmed their low values of dipole moment, i.e. 2.94 and 1.40 Debye, respectively (electronic supplementary material, figures S55 and S56) as compared with the key reference value of 4.80 D representing a pure charge of +1 and −1 separated by 100 pm. HOMO-LUMO gap and dipole moment findings aid both experimental and docking results. Overall the *in silico* studies are in agreement with the preliminary *in vitro* studies of the target chalcones.

## 6. Conclusion

The current study reports the synthesis of chalcone derivatives (**3a**–**q**) for their potential application as antibacterial and antileishmanial agents via *in vivo* and *in silico* analyses. The chemical structures of all the target chalcones were established by Fourier-transform infrared, NMR, and mass spectrometry spectroscopic techniques. Among all target chalcones, the derivative **3q** was noticed to be the most potent antibacterial and antileishmanial agent. The derivative **3i** exhibited the most potent antifungal activity. SAR analysis showed that the existence of amino moieties on ring-A and methoxy moieties on ring-B resulted in better antimicrobial activities of the target chalcones. The primary experimental results were also reinforced through the *in-silico* studies of the most potent chalcones (**3i** and **3q**). The *in vitro* and *in silico* data were found consistent. In general, the current investigations suggest that chalcones **3i** and **3q** can act as potential precursors for the discovery of new drug candidates to treat bacterial, fungal and leishmanial infections. Nonetheless, more experimental investigations are required to finally confirm their application in drug discovery.

## Data Availability

The datasets include the spectra obtained for the structural confirmation of the chalcones described in this manuscript and are stored as electronic supplementary material at Dryad [59]. The data are provided in electronic supplementary material [60].
